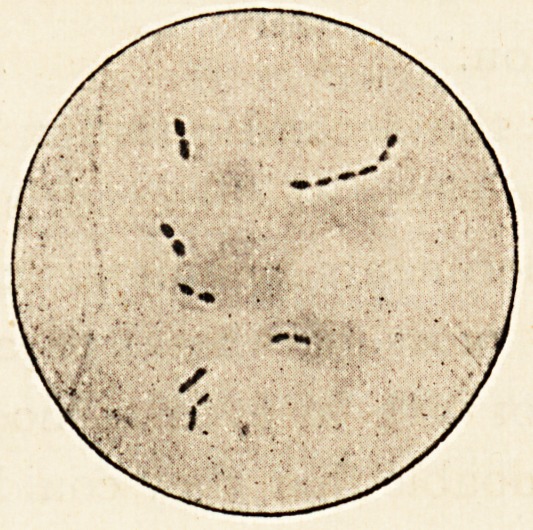# Pneumococci in the Urine

**Published:** 1895-06

**Authors:** G. Munro Smith

**Affiliations:** Professor of Physiology, University College, Bristol; Assistant-Surgeon to the Bristol Royal Infirmary


### PNEUMOCOCCI IN THE URINE.

BY

G. Munro Smith, M.R.C.S. Eng., L.R.C.P. Lond.,

*Professor of Physiology, University College, Bristol; Assistant-Surgeon to the Bristol Royal Infirmary.*

---

A lady, aged sixty, had an attack of croupous pneumonia in January last. It ran a favourable course, but on the seventh day from the commencement of the illness she complained of pain in micturition, and the urine became thick and ammoniacal. The bladder was washed out with solutions of boric acid, boro-glyceride, perchloride of mercury, &c., and iodoform bougies were used, but the cystitis persisted.

Some of the urine was drawn from her bladder into a clean vessel; and Dr. Davies and Dr. Dowson kindly examined the sediment, and prepared slides, from one of which the accompanying drawing was taken. Various microbes were found, but the most interesting were those having all the characteristic appearances of Fraenkel's pneumococcus. The preparation was stained by Gram's method, and it will be noticed that a clear capsule exists round the diplococci, which are in single pairs and in chains. The organism which is generally known as Fraenkel's pneumococcus was discovered by Sternberg and Pasteur in 1880. It was found by Fraenkel in his own sputum in 1885.

Talamon was the first to produce true croupous pneumonia by the injection of these germs into rabbits. They have a wide distribution, and have been found in ulcerative endocarditis, acute abscesses, cerebro-spinal meningitis, &c.

This case suggests two questions: (1) How did the microbes enter the bladder? and (2) Were they the cause of the cystitis? It has been strenuously maintained by Professor Guyon and others that infection of the bladder with germs is impossible without antecedent urethral disease or catheterisation.

In 1891 Dr. Bazy published cases of cystitis which supervened, without instrumentation or obstruction, during attacks of bronchitis and sore throats. In 1893 M. Emile Reymond inserted various micro-organisms into the peritoneal cavities of dogs and rabbits, and found that these germs travelled through the vesical walls into the bladder. Other cases of a similar nature have since appeared in print.[1]

It would appear, therefore, that these germs may enter the bladder by direct passage through the vesical walls from the peritoneal cavity, rectum, &c. It is interesting to note that the microbe most universally found in cystitis is the bacterium coli commune, whose principal *habitat* is the large intestine. There is evidence, also, that the bladder may be infected through the blood and through the kidney by what Jacobson calls "descending infection."

With regard to the second question suggested—viz., Were the pneumococci the cause of the cystitis?—no definite answer can be given. Experiments on animals could alone decide this. But inasmuch as most of these organisms, including the pneumococci, cannot live in even a moderately acid medium, it does not seem probable that they could exist long enough in the normal urine to effect any change in that fluid. The bacterium coli commune thrives, however, in an acid medium, and its constant presence in cystitis, coupled with this fact, would seem to show that it is the exciting cause of most cases of the disease.

[1] See, for example, *Progrès méd.*, tom. xix., 1894, p. 337.

## Figures and Tables

**Figure f1:**